# Effectiveness of intravitreal ranibizumab in exudative age-related macular degeneration (AMD): comparison between typical neovascular AMD and polypoidal choroidal vasculopathy over a 1 year follow-up

**DOI:** 10.1186/1471-2415-13-10

**Published:** 2013-04-04

**Authors:** Wataru Matsumiya, Shigeru Honda, Sentaro Kusuhara, Yasutomo Tsukahara, Akira Negi

**Affiliations:** 1 Department of Surgery, Division of Ophthalmology, Kobe University Graduate School of Medicine, 7-5-2 Kusunoki-cho, Chuo-ku, Kobe, 650-0017, Japan

**Keywords:** Intravitreal ranibizumab, Polypoidal choroidal vasculopathy, Typical neovascular age-related macular degeneration, One-year outcome

## Abstract

**Background:**

The effects of intravitreal ranibizumab (IVR) against exudative age-related macular degeneration (AMD) may be different associated with the lesion phenotype. This study was conducted to compare the outcomes of IVR between two different phenotypes of exudative AMD: typical neovascular AMD (tAMD) and polypoidal choroidal vasculopathy (PCV).

**Methods:**

This is a retrospective cohort study of 54 eyes from 54 subfoveal exudative AMD patients (tAMD 24, PCV 30 eyes). Three consecutive IVR treatments (0.5 mg) were performed every month, followed by re-injections as needed. Change in the best-corrected visual acuity (BCVA) and central retinal thickness (CRT) were then compared between the tAMD and PCV groups over 12 months of follow-up.

**Results:**

The mean BCVA was significantly improved (-0.11 logMAR units) at month 3 after the initial IVR (p <0 .001, Wilcoxon signed-rank test), and was sustained up to 12 months in all AMD patients (p =0.02). In the subgroup analysis, the tAMD group showed a significant improvement in their mean BCVA (-0.06, -0.17, -0.15 and -0.16 logMAR units at 1, 3, 6 and 12 months, respectively), but there was only a slight but non-significant improvement in the PCV group. The improvement in the BCVA was significantly greater in the tAMD group than in the PCV group (p = 0.043, repeated measures ANOVA) over 12 months. Both phenotypes showed significant improvements in the CRT during 12 months after the initial IVR.

**Conclusions:**

IVR is an effective therapy for tAMD and PCV in the BCVA improvement in Japanese patients over 12 months of follow-up. The phenotype of tAMD showed a significantly better outcome with IVR than PCV in terms of BCVA improvement.

## Background

The intravitreal injection of ranibizumab (IVR), an anti-vascular endothelial growth factor (VEGF) agent, is currently the choice of treatment for subfoveal choroidal neovascularization (CNV) due to age-related macular degeneration (AMD), which is a major cause of irreversible blindness in the elderly in industrialized countries [1,2]. Several studies from Western countries have reported significant improvements in the vision of AMD patients with predominantly classic, minimally classic and occult with no classic lesions. Two pivotal phaseIII clinical studies, the MARINA and ANCHOR studies [3,4], reported that VA improvements observed with IVR in the first 3 months were sustained and some additional improvement was seen over the trial period. EXTEND-I [5] in Japanese patients with CNV secondary to AMD also showed the comparable efficacy of IVR with the MARINA and ANCHOR studies. On the other hand, since this protocol was often not feasible, a customized/individualized pro re nata (PRN) dosing regimen of ranibizumab was investigated in order to achieve optimal VA results with fewer monthly injections. As a representative study of the PRN use of ranibizumab, the PrONTO study [6] proposed a flexible dosing regimen with the OCT and VA- guided criteria for re-treatment. These visual acuity results were similar to those from the MARINA and ANCHOR study [7]. However, the efficacy of IVR has not been well documented for exudative AMD in the Japanese population. A recent report described a good response to intravitreous bevacizumab, an anti-vascular endothelial growth factor (VEGF) antibody in off-label use, in Japanese AMD patients with classic CNV lesions, but there was limited efficacy in those with occult CNV lesions [8]. Those results might be attributed to the proportion of AMD subtypes in the Japanese population, which includes polypoidal choroidal vasculopathy (PCV) as the major phenotype of exudative AMD [9], and the effects of anti-VEGF therapy for PCV may differ from those for typical neovascular AMD (tAMD). Recent publications have reported that the effects of anti- VEGF therapy were limited in PCV [10,11], but it is not conclusive whether these phenotypes of AMD are associated with the effectiveness of IVR or not because there were only a few studies which conducted direct comparison in the outcomes of IVR between tAMD and PCV [12,13]. Since IVR is an expensive treatment and carries the risk of complications such as endophthalmitis, establishing precise indications for IVR is very important for the best benefit/risk ratio and cost performance of this therapy. The different effects of IVR against the different phenotypes may encourage a better selection of cases eligible for this therapy.

In this study, we first evaluated the efficacy of IVR in Japanese exudative AMD patients. Next, we performed a comparative assessment to determine whether the effects of IVR were different between tAMD and PCV in terms of visual acuity and the number of treatments over a 12 months follow-up period.

## Methods

This is a retrospective interventional cohort study of consecutive case series with exudative AMD treated by IVR. All cases in this study were Japanese individuals recruited from the Department of Ophthalmology at the Kobe University Hospital in Japan and IVR is a standard treatment for exudative AMD at the hospital. This study was approved by the Institutional Review Boards at the Kobe University Graduate School of Medicine, and was conducted in accordance with the Declaration of Helsinki. Written informed consent was obtained from all subjects.

Fifty-four eyes from 54 subfoveal exudative AMD patients (tAMD 24, PCV 30 eyes) who underwent the first IVR between April 2009 and April 2011, with an age over 50 years and the baseline visual acuity between 20/25 and 20/320 [14], were followed-up for 12 months. All patients received detailed ophthalmic examinations, including best-corrected visual acuity (BCVA) measurements, slit lamp biomicroscopy of their fundi, color fundus photography, fluorescein angiography (FA), indocyanine green angiography (ICGA) and optical coherence tomography (OCT). A detailed questionnaire on the patient’s basic characteristics including age, body weight and height, the presence or absence of hypertension and diabetic mellitus, and any history of smoking (current, past or non-smoker) was also obtained. The differential diagnoses of tAMD versus PCV were made in accordance with previous reports [15-17]. Briefly, the tAMD group included only exudative AMD with clear images of the vascular CNV networks on ICGA. In the present study, tAMD included both of classic CNV (15 eyes) and occult CNV (9 eyes), but not RAP lesion in accordance with the previous report [9]. The PCV cases in the present study showed subretinal reddish-orange protrusions corresponding to the choroidal origin of the polypoidal lesions, typically with the vascular networks in the posterior poles on ICGA [18,19]. Patients with past histories of retinal vessel occlusion, uveitis, rhegmatogenous retinal detachment or glaucoma were excluded. Patients who received previous photodynamic therapy (PDT) more than 3 months before IVR were included in the primary analysis to evaluate the effect of history of PDT.

In the present study, an intravitreal injection of 0.5 mg of ranibizumab in 0.05 ml was performed per treatment. All patients received 3 consecutive monthly IVRs, and were followed-up monthly for 12 months from the initial IVR in accordance with the PrONTO study criteria [6]. Additional IVRs were performed as needed, namely when sustained or recurrent serous retinal detachment, macular edema or hemorrhage was recognized by funduscopy or OCT. The FA and ICGA were repeated to evaluate the activities of the AMD lesions according to clinical recommendations if the funduscopic examinations could not explain the recent or progressive VA deterioration [7].

For statistical analysis, we first compared the gender, age, BCVA, greatest linear dimension (GLD), central retinal thickness (CRT), history of smoking and previous PDT therapy, presence of subretinal hemorrhage, serous retinal detachment, macular edema, retinal pigment epithelial detachment, subretinal fibrosis, hypertension and diabetes mellitus, and body mass index (BMI) at baseline between the tAMD and PCV groups. Changes in the BCVA and CRT were then compared within and between the tAMD and PCV groups until 12 months after the initial IVR. The visual acuities were determined using a Landolt C chart, and were converted to a logarithm of the minimum angle of resolution (logMAR) values for calculation. The CRT was obtained by OCT using the central 1 mm of the retinal thickness map with the Macular cube 200 × 200 scanning protocol (Cirrus HD OCT, Carl Zeiss Meditec, Dublin, CA). To evaluate the factors useful for predicting those patients whose BCVA at 12 months after the initial IVR were improved over the baseline BCVA, multivariate logistic regression analyses were performed using explanatory variables including gender, age, baseline BCVA, baseline GLD, baseline CRT, the number of previous PDT, history of smoking, presence of hypertension and diabetes mellitus, BMI and lesion phenotype (tAMD or PCV). Each variable was first calculated by a univariate logistic model, then the variables that showed some trend toward significant association (P value < 0.2) were further analyzed by a multivariate logistic model with stepwise method.

All statistical analyses were performed by MedCalc v.11.3 software (MedCalc Software, Mariakerke, Belgium). The chi-square test, paired t-test, unpaired t-test, Wilcoxon signed rank test or Mann-Whitney U test, whichever was the most appropriate, was performed to compare any two groups. A repeated measures analysis of variance (ANOVA) was used to make comparisons of changes in the BCVA between the tAMD and PCV groups over 12 months. P values of 0.05 or less were considered to be statistically significant.

## Results

**Figure 1:**
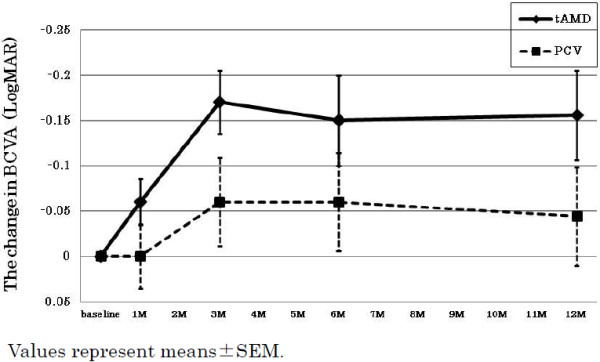
**Changes in the best corrected visual acuity (BCVA) for tAMD and PCV patients after intravitreal ranibizumab.** The BCVA was determined using the Landolt C chart, and was presented as decimal visual acuities. Diamonds with solid lines: tAMD; Squares with dashed lines: PCV. Values represent means ± SEM.

**Figure 2:**
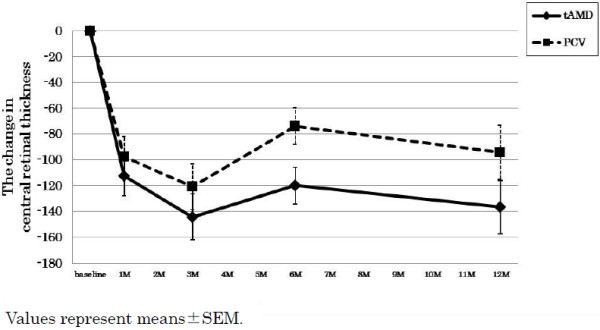
**Changes in central retinal thickness (CRT) for tAMD and PCV patients after intravitreal ranibizumab.** Diamonds with solid lines: tAMD; Squares with dashed lines: PCV. Values represent means ± SEM.

The data summary for each phenotype (tAMD and PCV) is shown in Table 1. None of the pre-treatment parameters were significantly different between any groups, except for a significantly larger GLD in the PCV group than in the tAMD group. In the time course analysis, the BCVA at 3 month after the initial IVR was significantly improved as compared with the baseline BCVA, and it was sustained for over 12 months in all AMD subjects (Table 2). In the subgroup analysis, the tAMD group showed a significant improvement in their mean BCVA compared to baseline (-0.06, -0.17, -0.15 and -0.16 logMAR units at 1, 3, 6 and 12 months, respectively) (Figure 1 and Table 2). In contrast, in the PCV group, the BCVA tended to improve over baseline (0.00, -0.06, -0.06 and -0.04 logMAR units at 1, 3, 6 and 12 months, respectively), but there was no statistical significance (Figure 1 and Table 2). A repeated measures ANOVA revealed a significantly better improvement of the BCVA in the tAMD group than in the PCV group at over 12 months (p = 0.043). In addition, the amplitude of the BCVA improvement was compared between tAMD and PCV at each time-point measured, which showed that tAMD achieved a significantly greater visual improvement than PCV at 3 M after the initial treatment (p = 0.43 at 1 M, p = 0.036 at 3 M, p = 0.066 at 6 M and p = 0.11 at 12 M). To exclude a possible influence of previous treatment by PDT, we evaluated the visual outcome of IVR for 16 tAMD patients and 18 PCV patients who were treatment naïve. In this analysis, the mean BCVA were significantly improved over baseline in tAMD group (-0.07, -0.18, -0.17 and -0.14 logMAR units at 1, 3, 6 and 12 months, p = 0.055, 0.001, 0.008 and 0.049, respectively; Wilcoxon signed rank test), although PCV group showed modest and non-significant improvement in their mean BCVA over 12 months of follow-up (-0.02, -0.08, -0.07 and -0.07 logMAR units at 1, 3, 6 and 12 months, p = 0.31, 0.07, 0.07 and 0.23, respectively). The time course of the mean CRT in the tAMD and PCV groups is shown in Figure 2 and Table 2. Both phenotypes showed significant improvements in the CRT over 12 months after the initial IVR. There were no significant differences in the improvements of the CRT in the tAMD versus the PCV group. The number of re-treatments after the initial 3 consecutive injections was 0.9 ± 0.9 (mean ± SD) in the tAMD group, and 1.2 ± 1.3 in the PCV group, which was not significantly different (p = 0.32, unpaired t-test)

**Table 1:** Summary of the participants stratified by AMD phenotype

	**tAMD (n = 24)**	**PCV (n = 30)**	**Total (n = 54)**	**P value**
Eye (right eye%)	6 (25%)	13 (43%)	19 (35%)	0.26✝
Gender (males%)	18 (75%)	21 (70%)	39 (72%)	0.92✝
Age, Mean (±SD) (years)	73.2 (±8.5)	76.0 (±7.6)	74.7 (±8.1)	0.21*
Baseline Vision LogMAR (±SD)	0.60 (±0.28)	0.47 (±0.26)	0.53 (±0.28)	0.09*
Baseline macular thickness (±SD) (μm)	392 (±141)	352 (±119)	369 (±129)	0.27*
Baseline GLD (±SD) (μm)	3610 (±962)	4650 (±1665)	4188 (±1480)	<0.01*
BMI (±SD)	22.6 (±4.0)	22.1 (±2.8)	22.4 (±3.4)	0.62*
Present or ever-smoking	17 (71%)	20 (67%)	37 (69%)	0.97✝
Hypertension	11 (46%)	13 (43%)	24 (44%)	0.92✝
Diabetic mellitus	5 (21%)	5 (17%)	10 (19%)	0.97✝
Previous PDT therapy				0.40✝
0	16 (67%)	18 (60%)	34 (63%)	
1	5 (21%)	4 (13%)	9 (17%)	
2≦	3 (12%)	8 (27%)	11 (20%)	
Subretinal hemorrhage (>1DA)	4 (17%)	8 (27%)	12 (22%)	0.58✝
Serous retinal detachment	18 (75%)	26 (87%)	44 (81%)	0.46✝
Macular edema	11 (46%)	12 (40%)	23 (43%)	0.88✝
Retinal pigmented epithelium detachment	21 (88%)	30 (100%)	51 (94%)	0.16✝
Subretinal fibrosis	7 (29%)	4 (13%)	11 (20%)	0.27✝

*GLD,* Greatest linear dimension; *BCVA,* Best corrected visual acuity; *CRT,* Central retinal thickness; *BMI,* Body mass index; * unpaired *t*-test ✝ Chi-square test.

**Table 2:** Changes in the BCVA and CRT from baseline after IVR treatment in all AMD, tAMD and PCV patients

	**1 Month**		P value*	**3 Months**		P value*	**6 Months**		P value*	**12 Months**		P value*
**Change in BCVA**	Mean	Median (IQR)		Mean	Median (IQR)		Mean	Median (IQR)		Mean	Median (IQR)	
total	−0.03	0.00 (-0.12 - 0.00)	0.12	−0.11	−0.12 ( -0.24 - 0.00)	<0.001	−0.10	−0.12 ( -0.24 - 0.00)	0.001	−0.09	−0.12 (-0.30 - 0.00)	0.02
tAMD	−0.06	0.00 (-0.17 - 0.00)	0.01	−0.17	−0.20 (-0.30 - -0.05)	<0.001	−0.15	−0.18 (-0.30 - -0.05)	0.006	−0.16	−0.18 (-0.30 - -0.05)	0.01
PCV	0.00	0.00 (-0.10 - 0.00)	0.73	−0.06	−0.07(-0.22 - 0.00)	0.08	−0.06	−0.04 (-0.18 - 0.00)	0.08	−0.04	−0.05 (-0.24 - 0.10)	0.33
**Change in CRT**	Mean	Median (IQR)		Mean	Median (IQR)		Mean	Median (IQR)		Mean	Median (IQR)	
total	−104	−74 (-142 - -25)	< 0.001	−132	−119 (-170 - -47)	< 0.001	−95	−53 (-126 - -28)	< 0.001	−94	−80 (-140 - -40)	< 0.001
tAMD	−112	−72 (-147 - -29)	< 0.001	−144	−93 (-191 - -72)	< 0.001	−120	−52 (-154 - -32)	< 0.001	−137	−97 (-197 - -52)	< 0.001
PCV	−98	−107 (-142 - -25)	< 0.001	−121	−128 (-170 - -45)	< 0.001	−74	−55 (-119 - -28)	< 0.001	−94	−71 (-133 - -33)	< 0.001

*BCVA*, Best corrected visual acuity (LogMAR); *CRT*, Central retinal thickness; *IQR,* Interquartile range.

*Wilcoxon signed rank test (comparison with baseline).

The results of the stepwise logistic regression analyses are shown in Table 3. The lesion phenotype (tAMD vs. PCV) was an independent prognostic factor significantly associated with the improvement in the BCVA at 12 months after the initial IVR, which was actually better in the tAMD group than in the PCV group.

**Table 3:** Results of stepwise logistic regression analyses of preoperative variables associated with an vision gainer (improvement in the BCVA >0 LogMAR units from the baseline) at 12 months after the initial IVR

**Variable**	**OR**	**95% CI**	**P value**
BMI	1.3	1.0 - 1.6	0.031
Phenotype (PCV = 0, tAMD =1)	4.3	1.2 - 15.9	0.029

*OR,* Odds ratio; *CI,* Confidence interval; *BMI,* Body mass index.

The number of vision gainer and non-gainer was 34 and 20.

No ocular or systemic complications were detected during the follow-up in the present study.

## Discussion

We investigated the 12 month outcomes of IVR in Japanese AMD patients, and investigated whether the different phenotypes of exudative AMD influenced the visual outcomes of IVR. Our results demonstrated that IVR improved the mean BCVA of exudative AMD in the Japanese population, and the visual improvement was significantly greater in the tAMD subjects than in the PCV subjects. The phenotype of exudative AMD was a possible significant prognostic factor for the visual acuity after IVR.

Currently, IVR is the leading therapy for exudative AMD since the ANCHOR study demonstrated significantly better visual outcomes with monthly IVR than PDT in exudative AMD patients (most of them were likely tAMD patients) in Western countries [20,21]. However, in Japanese exudative AMD, which likely includes a number of PCV patients, IVR may not show as good an outcome as in Caucasian subjects [8,9,22]. In the present study, the mean and median LogMAR visual acuity of all AMD were significantly improved by -0.09 and -0.12 logMAR units at 12 months post-initial IVR, respectively. This result was compatible to the SUSTAIN study [23] and a previous report [24], but lower than the PrONTO and CATT study. In addition, our results showed a significant increase in the mean BCVA in patients with tAMD, and a modest improvement in those with PCV. On the other hand, Song et al. [25] reported that IVR without PDT for PCV in Korean patients resulted in visual and anatomical significant improvements over a 1-year follow-up period.

PCV is known to have different characteristics as compared with tAMD, such as orange-red protrusions at the posterior pole of the retina and distinct forms of choroidal vascular abnormalities, including vascular networks of choroidal origin with polypoidal lesions at their border visualized by ICG [16,18]. In addition, Nakashizuka et al. [26] suggested that the histopathologic characteristic of PCV was hyalinization of the choroidal vessels like arteriosclerosis, which is different from the CNV associated with tAMD. Since PCV accounts for 54.7% of patients with neovascular AMD in the Japanese population [15] and 22.3% in the Chinese population [27], it is important to determine if there are some differences in the efficacy of anti-VEGF therapy against PCV and tAMD to choose the correct intervention for neovascular AMD in Asian populations.

It was interesting that the PCV patients showed poorer improvements in their BCVA than tAMD patients, although both phenotypes showed similar and significant improvements in their CRT during the 12 months after the initial IVR. A previous report showed a decrease in macular edema after three monthly bevacizumab injections in PCV cases [28]. Similarly, the macular edema evaluated by the CRT measurements was improved in four out of five eyes with PCV (80%) in the PEARL study. However, the improvement in the BCVA was less than that in the ANCHOR or MARINA trials, although the reasons are unknown [29]. We hypothesized that there might be factors other than macular edema which influence the visual acuity in tAMD and PCV cases differently. Although the mean baseline GLD was significantly greater in the PCV group than in the tAMD group in the present study, the results of the multivariate logistic regression analysis revealed that the lesion phenotype (tAMD or PCV) was the independent prognostic factor for the 12 month visual outcome after IVR. Moreover, the ANOVA indicated a significantly better visual prognosis in tAMD than PCV treated by IVR over a 12 month follow-up period. The logistic regression analysis also determined higher BMI as an independent beneficial factor for visual improvement after IVR, although the reason was unknown. A recent study demonstrated that circulating VEGF-A levels were strongly correlated with BMI [30] and the patients with higher baseline VEGF-A levels might be more susceptible to ranibizumab treatment. In the present study, the mean number of IVR injections was 4.1 (3.9 in tAMD group, 4.2 in PCV group), which was compatible with previous reports [24,31]. However, these reports and our results did not achieve an equivalent improvement in BCVA when compared to the ANCHOR or MARINA study. This suggests that some patients in our study might have been under-treated by the PRN protocol, as discussed in the SUSTAIN study [23]. In addition, there might be an extended interval between a decision of retreatment and a performance of retreatment in our study. Delay of treatment might have a potential risk of irreversible VA deterioration [32], and may be associated with a lower number of retreatments with IVR.

The effects of IVR against PCV are currently contentious, and no consensus has been made to date. Some studies reported that the polypoidal lesions of PCV were barely resolved by anti-VEGF monotherapy, which might explain the limited efficacy of IVR against PCV [9,10]. However, other reports suggested that the disappearance of the polypoidal lesions occurred at a high rate in PCV cases with anti-VEGF monotherapy [26,33,34]. Although the PCV cases showed a modest improvement in their mean BCVA over 12 months in the present study, our results demonstrated that IVR rescued the vision of PCV patients, since we previously reported that the mean BCVA of PCV cases had deteriorated significantly by 12 months following their natural course [35]. In addition, some recent studies reported a significant improvement of the visual acuity of PCV patients using IVR [26,33,34]. A recent report demonstrated that several pretreatment factors of PCV influenced the outcome of IVR [36], which might cause the inconsistent results of those studies regarding the effect of IVR on PCV. The worse baseline BCVA in the tAMD group than the PCV group (though not significant) might be associated with the Ceiling effects on the BCVA change after the treatment in the present study [37]. Further studies with prospective nature using larger and better matched populations will be required to make a robust conclusion. The lack of data about the duration of symptoms that may influence the outcome of the treatment is another limitation of the present study.

It is interesting that several reports have shown different outcomes for PDT between tAMD and PCV patients [38,39]. In those reports, significantly better visual outcomes were demonstrated for PCV than tAMD. Recently, a multi-center study was conducted to compare the effects of PDT and IVR in patients with PCV, and reported that both therapies as well as their combination (PDT + IVR) resulted in improvements of the patients’ visual acuity at 6 months post-treatment [40]. In addition, PDT monotherapy and combination therapy achieved a significantly higher proportion of patients with complete polyp regression at 6 months than IVR monotherapy. Hence, it is important to evaluate the long-term results of IVR with a large number of subjects to determine the efficacy and durability of this therapy, particularly in PCV patients. Taken together, further investigation will be needed to determine the correct indications for IVR for exudative AMD.

## Conclusion

In conclusion, IVR is an effective therapy to improve the vision of Japanese AMD patients, although this effect might be greater in tAMD patients than in PCV patients.

## Competing interest

No authors have any competing interest to disclose.

## Authors’ contributions

WM carried out the data analysis and drafted the manuscript. SH conceived of the study and participated in the design of the study, acquired the data and participated in the data analysis. SK participated in acquisition, analysis and interpretation of the data. YT participated in the acquisition and interpretation of the data. AN participated in interpretation of the data and helped to draft the manuscript revising it critically for important intellectual content. All authors read and approved the final manuscript.

## Funding

This study was supported by a Grant-in Aid (C) 23592567 from the Ministry of Education, Science and Culture, Tokyo, Japan (S.H.), and by a grant from the Takeda Science Foundation, Osaka, Japan (S.H.). The funding organization had no role in the design or conduct of this research.

## Pre-publication history

The pre-publication history for this paper can be accessed here:

http://www.biomedcentral.com/1471-2415/13/10/prepub
